# Obstructive Sleep Apnea: A Look towards Micro-RNAs as Biomarkers of the Future

**DOI:** 10.3390/biology12010066

**Published:** 2022-12-30

**Authors:** Giorgia Moriondo, Piera Soccio, Pasquale Tondo, Giulia Scioscia, Roberto Sabato, Maria Pia Foschino Barbaro, Donato Lacedonia

**Affiliations:** 1 Department of Medical and Surgical Sciences, University of Foggia, 71122 Foggia, Italy; 2 Institute of Respiratory Diseases, Policlinico Foggia University Hospital, 71122 Foggia, Italy

**Keywords:** obstructive sleep apnea (OSA), intermittent hypoxia, cancer, micro-RNA, biomarkers

## Abstract

**Simple Summary:**

Intermittent hypoxia associated with obstructive sleep apnea is often associated with cardiac, metabolic, and neoplastic alterations. A large number of studies in recent years have demonstrated that microRNAs play an essential role in the pathogenesis of obstructive sleep apnea and offer considerable potential as a critical new target for the diagnosis and management of patients with this disease. In this review, we highlight the different roles of microRNAs in obstructive sleep apnea and identify their regulatory roles in disease development and progression.

**Abstract:**

Sleep-disordered breathing (SDB) includes a broad spectrum of diseases, of which obstructive sleep apnea syndrome (OSA) is the most clinically significant manifestation. OSA is a respiratory disorder characterized by episodes of complete or partial obstruction of the upper airways that disturb ventilation and sleep architecture. In recent years, interest in the clinical implications of OSA seems to have increased, probably due to the numerous studies that have shown the existence of an important correlation between OSA and cardiovascular, dysmetabolic, and neoplastic changes. The guidelines currently available highlight the importance of diagnosis and effective treatment for OSA, underlining the need for new biomarkers that are useful in clinical practice, feasible, and reproducible to guide medical decision making. In this review, we intend to provide an overview of the potential role of microRNAs as new indicators for OSA management. MicroRNAs (miRNAs) are small non-coding RNA molecules that play an important role in RNA silencing and regulation of gene expression at the post-transcriptional level. These can bind specifically to their target genes by forming silencing complexes, thus inducing degradation or altered gene expression. A wide range of miRNAs have been extensively studied in a variety of diseases including cancer, and recently, miRNAs have been shown to have enormous potential to function as diagnostic and clinical biomarkers of disease. This review includes recent studies that establish the inevitable role of miRNAs in the pathogenesis of OSA.

## 1. Obstructive Sleep Apnea

Obstructive sleep apnea (OSA) is the most common sleep-disordered breathing (SDB), characterized by collapse complete or partial of the upper airways during sleep, leading to respiratory alteration of airflow [1]. This phenomenon causes alterations in the structure of sleep (sleep fragmentation), alterations in gas exchange (oxygen/carbon dioxide), and hemodynamic and cardiovascular alterations (changes in heart rate, increase in blood pressure, cardiac arrhythmias, etc.), which can persist even in the waking hours.

It is difficult to have a reliable estimate of the prevalence of OSA due to the different criteria used for diagnosis and the low number of patients examined. However, a recent study estimates that OSA is a pathological condition with a high epidemiological impact; in fact, nearly one billion of individuals are affected by this condition worldwide [2,3,4]. Population studies have shown that both sleep apnea and daytime sleepiness are frequent in the general population and increase with age. The main risk factors for OSA in adults are sex, obesity, and age; they also seem to constitute factors favoring genetic predisposition and the alteration of the muscle tone of the upper airways that occurs physiologically during sleep. Women are relatively protected from the onset of obstructive sleep apnea during the fertile age, presenting a risk substantially comparable to men in the post-menopausal period [5,6]. In children, the main risk factor is adeno-tonsillar hypertrophy [7].

It is now known that sleep is not just a passive phenomenon. In fact, during sleep, the central nervous system (CNS) performs numerous activities although it maintains a physiological state of loss of consciousness and poor responsiveness to stimuli.

In humans, normal sleep has its own rhythm composed of two distinct states, namely NREM (non-rapid eye movement) and REM (rapid eye movement or paradoxical sleep), during which there is a cyclic sequence of frequencies waveforms that can be observed on the electro-encephalogram (EEG) during the polysomnographic examination. This composition of NREM + REM sleep is called the sleep cycle. In a healthy adult, four to five sleep cycles usually occur each night [8].

Conversely, in subjects with OSA, the repetitive episodes of partial (hypopnea) or total (apnea) upper airway obstruction result in an alteration of sleep architecture [9].

In general, OSA-related respiratory events may worsen during REM sleep because there is muscular atonia in this sleep stage, and therefore, it is easier to observe the collapse of the upper airways. Most of the apnea events lead to the awakening of the subject and the return of muscle tone with consequent cessation of the airway obstruction generating a continuous fragmentation of sleep. The reduction of alveolar ventilation involves a reduction in the partial pressure of oxygen and an increase in the partial pressure of CO_2_, which stimulate the chemoreceptors and induce activation of the sympathetic nervous system; it is believed that it is this stimulus that determines the transition to a more superficial phase of sleep until actual awakening. With the end of the obstructive episode and the resumption of ventilation, this neuro-hormonal stimulus, associated with the alteration of ventilatory mechanics, determines the passage towards a more superficial phase of sleep or a short awakening, which involves a fragmentation of night sleep that is more or less evident and inconstantly perceived subjectively by patients.

OSA severity is generally expressed by the apnea-hypopnea index (AHI, apnea-hypopnea index) with three severity degrees: mild if the AHI is between 5 and 15; moderate if the AHI is between 15 and 30; severe if the AHI is greater than 30 events·h^−1^. Nonetheless, other factors such as oxyhemoglobin desaturation and the percentage of time in which desaturation persists during sleep may also influence the severity of the disease [9].

However, it is necessary to specify that, despite the ease of diagnosis, patients with OSA show different clinical presentations; there are symptomatic, asymptomatic, or minimally symptomatic patients, and these different phenotypic profiles can have a significant impact on clinical outcomes. In fact, the prognosis of symptomatic or asymptomatic patients with OSA is not necessarily the same even considering a similar severity of OSA [10].

The symptoms that individuals with OSA most frequently report include: excessive daytime sleepiness, reduced attention span and concentration, lower cognitive performance, and tendency to doze off in unsuitable socio-work circumstances and during the performance of actions perceived as monotonous or repetitive or scarce in stimuli, such as driving a car along a route that does not require the subject’s alertness. Nocturia and decreased libido are also reported. The main symptoms and signs of the syndrome are shown in Table 1.

Many factors can contribute to the development of OSA. Among the most important are the following:

(1) Anatomical changes that can contribute to the reduction of the oropharyngeal space [12,13]. Therefore, in obese subjects, where the neck circumference increases (>43 cm) and craniofacial alterations are often present, the risk of developing the disease increases considerably;
(2) Smoking can contribute to upper airway dysfunction, as it promotes relaxation of the airway muscles and causes neural reflexes due to the nicotine [14,15];
(3) Sleeping in the supine position facilitates the onset of apnea;
(4) The presence of craniofacial anomalies (retrognathia and micrognathia, angulation of the skull base), nasal obstructions, tonsillar and/or adenoid hypertrophy, ogival palate, prolapse of the uvula, macroglossia, or edema of the larynx [14];
(5) The use of alcohol or other substances such as muscle relaxants or sedatives [16].

There are numerous validated anamnestic tools that can be useful for an initial evaluation of the symptoms/signs and risk factors related to the pathology of sleep apnea; however, the diagnostic gold-standard investigation is the overnight full-polysomnography (PSG), an investigation that takes place at night while the patient is asleep and which provides an assessment of the severity of the syndrome allowing the determination of the type of apnea (obstructive, central, or mixed) through the monitoring of various physiological and pathological parameters, such as the apnea index and hypopnea, oxyhemoglobin saturation, awakenings and micro-awakenings, postural changes, the distribution of sleep stages, the electrocardiographic record, and the intensity and frequency of snoring [6,17]. However, PSG is a complex investigation that requires trained technical personnel and is expensive, and it also requires long waiting times. For this reason, portable devices have been developed over the years that are cheaper than PSG to perform sleep investigations; although these devices require trained technical personnel to instruct the patient, set up/download the equipment, and analyze the data, they are easier to manage than a standard PSG and can be used at home with the large benefit of reducing waiting lists for a sleep investigation [18,19].

Currently, there are no effective pharmacological treatments for this pathology [20]. Weight loss and change of posture during sleep are the first precautions to be implemented for these patients. Continuous Positive Airway Pressure devices (CPAP) represent the first-line treatment for patients with OSA [21]. They compress the ambient air and channel it at a given pressure into a nasal or oro-nasal mask in order to ensure the patency of the upper airways. The amount of pressure must be established individually for each patient, taking into account the fact that the optimal pressure is the minimum required to completely eliminate obstructive apneas, hypopneas, and snoring. Be that as it may, there are other therapeutic approaches, such as surgical treatment, useful especially for patients with craniofacial or pharyngeal abnormalities.

The clinical implications of obstructive sleep apnea are the subject of increasing interest in recent years, also based on the results of numerous studies that have shown the existence of an important correlation between this disorder and cardiovascular, dysmetabolic, and neurohormonal disorders [22] and cancer [23]. OSA, through sleep fragmentation and intermittent hypoxia, involves sympathetic activation, endothelial dysfunction, hypercoagulative state, inflammatory state, and oxidative stress with multi-organ involvement and possible and important comorbidities, in particular, cardiovascular, cerebrovascular and neoplastic.

Among the various comorbidities associated with sleep apnea, cardiovascular ones play a role of primary importance and are mainly due to the activation of the sympathetic nervous system, endothelial dysfunction, hypercoagulation, and oxidative stress. In fact, in patients with OSA, hypertension (often drug-resistant) [24], stroke, heart attack, congestive heart failure, coronary heart disease, atrial fibrillation, and other forms of cardiac arrhythmias are very frequent [22].

Systemic arterial hypertension is found in 40–60% of patients with this condition, and often, individuals with moderate-to-severe OSA are more likely to develop acute myocardial infarction. Severe OSA also increases the chance of developing cardiac arrhythmias three to four times and increases the chance of stroke by 3.8 times [22,25].

In addition, it seems that OSA may contribute to the development of metabolic alterations such as diabetes mellitus (type I and II), metabolic syndrome, and non-alcoholic fatty liver disease [26]. Excessive daytime sleepiness (EDS), cognitive impairment, learning deficits, and mental disorders (depression and anxiety) are other disorders often associated with OSA. In fact, it is believed that about 50% of OSA patients develop depression [27]. The chronic reduction of oxygen concentration in the blood, consequent to OSA, generates an inflammatory cascade and oxidative stress mediators that cause diffused vascular epithelial damage [28]. A possible role of intermittent hypoxia has recently emerged as an independent neoplastic risk factor as well as a potential worsening factor in the response to cancer therapies and in the level of neoplastic aggression [23,29,30].

Based on what has been said so far, it is easy to understand that diagnosis is a fundamental step in the management of this disease; however, the systems currently used to diagnose OSA require nocturnal recordings as well as lengthy and expensive procedures. The costs for the PSG are in fact high, and the dedicated centers are not numerous. In this regard, the need to find biomarkers that are useful in clinical practice and that are feasible and reproducible to help doctors in the management of these patients is evident. New methods of early diagnosis of the disease must be used as the first screening and follow-up tool for subjects at risk. The aim of this review is therefore to explore microRNAs as potential new biomarkers for the diagnosis of OSA not only to improve the current knowledge of the molecular pathways underlying this pathology but also to find new markers capable of predicting the different complications that these subjects can encounter.

## 2. Methods

Relevant articles published between 1993 and December 2022 from *PubMed*, *Embase,* and *Web of Science* were screened. A narrative review of previously published studies was conducted to evaluate the effect of OSA and, in particular, of IH on the expression of various microRNAs and the OSA-cancer correlation. The search term used included OSA, sleep apnea, intermittent hypoxia, cancer, microRNA, and biomarkers.

Two investigators (G.M. and P.S.) independently screened all references for inclusion, and discrepancies were resolved by discussion among the team. Eligibility was considered for items that had a control group, exhibited results with OSA, and showed a relationship between miRNA and IH. No language restrictions were applied. The full texts of all studies referring to OSA, IH, miRNA, and cancer were reviewed for final inclusion.

**Figure 1:**
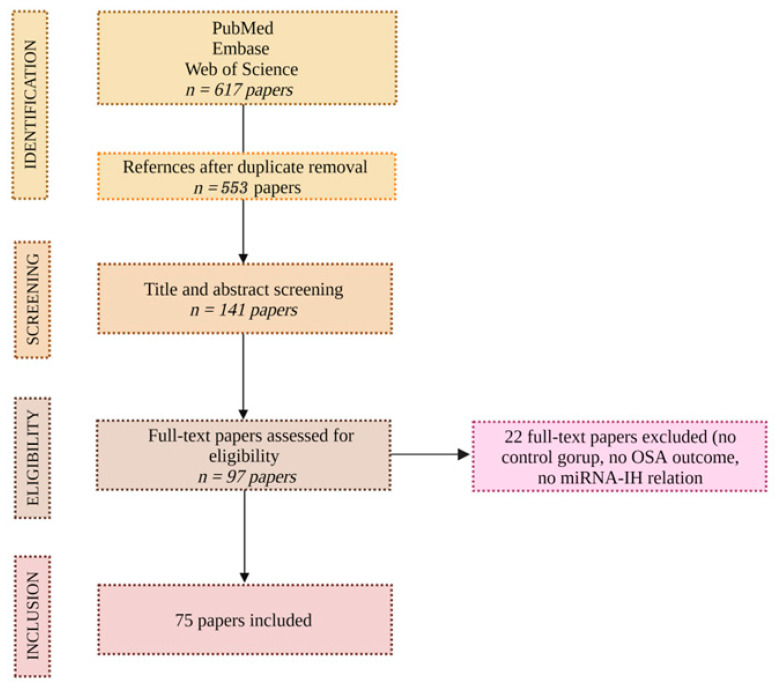
CONSORT diagram.

Based on the above, 617 articles were reviewed, and 75 were included. The investigators extracted the data and inspected each reference identified by the search and applied inclusion criteria. In cases where the same studies were reported in more than one publication, the study’s results were accounted for only once. The consort diagram in Figure 1 exhibits the above.

## 3. OSA and Intermittent Hypoxia

Obstructive sleep apnea syndrome is a disease characterized by repeated episodes of apnea and hypopnea during sleep, which induce cyclical changes in oxyhemoglobin saturation/desaturation and sleep fragmentation. Intermittent hypoxia (IH) is the main pathophysiological mechanism in OSA [31].

IH associated with OSA is characterized by cycles of hypoxia and reoxygenation that are markedly different from those of continuous hypoxia. The substantial difference between the two types of hypoxia is due to the oxygenation pattern (fluctuating or chronically low) and the length and frequency of hypoxic periods. However, there are also differences in cellular and molecular response at both the systemic and local levels. These differences are critical for assessing the effects of downstream disease and for predicting response to specific therapies [29].

There are no consistent definitions in the literature to describe intermittent and chronic hypoxia. Nevertheless, we know that in conditions of intermittent hypoxia, the duration of the hypoxia/reoxygenation cycles can vary by several orders of magnitude (from seconds to hours), and also, the number of cycles (over a period of minutes, hours, days, or weeks) can be different.

IH, associated with OSA, promotes oxidative stress by increasing the production of reactive oxygen species (ROS), increases angiogenesis, induces greater sympathetic activation with increased blood pressure, and generates systemic and vascular inflammation with endothelial dysfunction that contributes to the various co-morbidities found in patients with OSA.

In hypoxic conditions, the body implements a series of coordinated responses aimed at restoring ideal levels of O_2_ [32,33]. If the hypoxia is severe and prolonged, a series of factors are activated that can lead to cell death. On the other hand, if the hypoxia is moderate and intermittent, adaptation mechanisms are activated that last over time.

One of the responses at the molecular level consists in the activation of specific, i.e., hypoxia-inducible, transcription factors called HIFs (hypoxia inducible factors) [32] and their second messengers, erythropoietin (EPO), and the growth factor for the vascular endothelium (VEGF).

HIF are hetero-dimeric complexes composed of an α subunit, whose stability is oxygen-dependent, and an oxygen-independent β-subunit. Three α subunits (1α, 2α, and 3α) and one β subunit (also called ARNT, aryl-hydrocarbon receptor nuclear translocator) have been identified, which, however, have different variants of splicing [32,33].

In the presence of normal quantities of oxygen, in the cytoplasm, the α subunits undergo two hydroxylation events catalyzed by the enzymes PHDs (prolyl hydroxylase domain proteins) and FIH (factor inhibiting HIF), which cause their degradation mediated by proteasome [30]. In hypoxic conditions, however, the PHDs and FIH enzymes are inactive due to the lack of oxygen necessary for hydroxylation reactions. In this way, the α subunit is stabilized and translocates in the nucleus to bind to the β subunit, forming a heterodimer (HIFα/HIFβ), which represents the active form of HIF, able to function as a transcription factor. The identified HIFs (HIF1α/HIFβ, HIF2α/HIFβ, and HIF3α/HIFβ) become active and therefore functional under hypoxic conditions. HIFα/HIFβ (HIF) heterodimer binds to a region of DNA from the identified consensus nucleotide sequence (5′-RCGTG-3′), called HRE (hypoxia response element, or HIF binding site), present in the promoters of hypoxia-inducible genes [34].

HIF-1α is a small molecule, present in all cells, which regulates the presence of oxygen at the cellular level and stimulates the cell nucleus to produce factors that increase the presence of oxygen as a “countermeasure” to hypoxia.

The first major target of HIF-1α is EPO [35]. EPO is synthesized by cells in response to hypoxic conditions, increases the oxygen-carrying capacity of the blood by stimulating erythropoiesis in the bone marrow, and represents one of the first indicative markers of hypoxia. Secondly, HIF-1α induces the expression of the vascular endothelial growth factor. VEGF is a potent mediator of angiogenesis that produces multiple effects, including those related to the development and physiology of the lungs [35].

Nitric oxide synthase (NOS) and heme oxygenase (HO) are also two HIF-1α target genes, both of which activate vasodilation, increasing local blood flow and lowering blood pressure.

Fourthly, anaerobic glycolysis becomes the predominant form of cellular ATP generation under conditions of limited oxygen supply. Therefore, glucose uptake and glycolysis are upregulated by HIF-1α.

In addition, HIF-1α, in hypoxic conditions, increases the expression of tyrosine hydroxylase, the rate-limiting enzyme for dopamine biosynthesis.

Finally, iron metabolism in humans is also stimulated in hypoxic circumstances, in particular, in the presence of factors such as ceruloplasmin, transferrin, and transferrin receptor [36].

Iron is a vital element in all living organisms and is required as an essential cofactor for oxygen-binding proteins. Extensive research has shown that HIF transcription factors function as central mediators, allowing cells to adapt to critically low oxygen levels in both normal and compromised tissues.

HIF-1, therefore, regulates the transcription of numerous genes, many of which encode proteins involved in the metabolic adaptation to hypoxia.

To date, more than 60 genes have been identified as presumed targets of HIF-1, among which we find genes involved in the development and progression of cancer, angiogenic factors, proliferation and survival factors, glucose transporters, and glycolytic enzymes.

HIF-1α has been found to be expressed to a greater extent in many solid tumors. This feature can be explained precisely by its ability to induce the expression of a wide spectrum of genes that allow survival in hypoxic conditions. It is important to underline that HIF-1α is activated by many oncogenic pathways such as PI3K or RAS, the mechanisms of which are not well-known. The central role of HIF-1α in tumor pathogenesis is thus evident [23,29,30].

## 4. OSA and Cancer

The fundamental pathophysiological mechanism that characterizes OSA is represented by chronic intermittent nocturnal hypoxia, which generates a chronic inflammatory cascade that causes diffused vascular epithelial damage [37]; the same chronic inflammatory mechanism could promote the development of cancer through the release of angiogenic factors.

A recent study showed, in a mouse model of OSA, the possibility of developing malignant tumors towards blood or skin cells (malignant melanoma) by triggering mechanisms of systemic inflammation, oxidative stress, and immune dysregulation [38], which are all factors that have been associated with oncogenesis.

These data allowed us to hypothesize that exposure to chronic intermittent nocturnal hypoxia in humans could determine the origin of the malignant cell neoformation and increase the progression and distant metastatic spread [39,40].

In this regard, numerous clinical studies have been conducted in humans to evaluate the possible relationship between OSA and cancer.

Initially, an analysis conducted on 1522 patients found that subjects with severe OSA were at almost five times the risk of developing cancer [41].

Subsequently, a Spanish study conducted on about 5000 patients with OSA showed that the severity of the disease expressed by the intensity of nocturnal hypoxemia has a strong link with the development of malignant neoplasms in various parts of the body [42].

This relationship was also highlighted by a Canadian retrospective cohort study that showed that there is a close relationship between OSA and cancer [43].

In addition, according to a study conducted by Hakim et al., disturbed and discontinuous sleep troubled by frequent awakenings could worsen the prognosis of tumors, increasing their aggressiveness and the ability to invade surrounding tissues. The mechanism would depend on the immune system, which, in the presence of irregular sleep, would not be able to effectively fight the disease [44]. The investigation was conducted using two groups of mice with tumors, half of which were placed in special cages capable of simulating the effects of restless sleep and the other half in normal cages. After four weeks, tumors developed by mice housed in cages simulating sleep disturbances were twice as large as tumors from mice that slept continuously. Continuing in a new series of experiments, the researchers showed that these tumors are also much more aggressive, identifying a link of this phenomenon with certain cells of the immune system called tumor-associated macrophages. There are two types of these cells: TM1 that induces a strong immune response against cancer and TM2 that instead creates a fertile ground for the spread of the tumor, increasing its vascularization and therefore promoting its growth. Analyzing the tumors developed in the presence of restless sleep, the researchers discovered the presence in very high quantities of TM2 associated with high levels of a molecule called TLR4 (Toll-like receptor 4). At this point, the researchers replicated the experiment by injecting cancer cells into mice that were genetically modified to not express the TLR4 molecule; as a result of the new experiment, the differences in the development of neoplasms between animals with sleep disorders and those who slept regularly disappeared completely.

A further study by Torres M. et al. hypothesized a link between OSA and the development of lung cancer. Observing the laboratory mice, the researchers have shown that the lack of oxygen intermittently during sleep accelerates the growth of lung cancer but only in younger subjects, possibly due to a different response of macrophages and tumor-associated lymphocytes [45].

Recently, a study conducted on 19,000 patients suffering from obstructive sleep apnea found a correlation between this disorder and the diagnoses of breast and prostate cancer, showing that the probability of a double diagnosis—OSA and cancer—is higher in women [46]. The researchers analyzed data from 19,556 people included in the European database on sleep apnea (ESADA) to explore the link between the severity of this disorder, low blood oxygen levels, and cancer development. Participants included 5789 women and 13,767 men, and for each participant, age, body mass index, smoking, and alcohol consumption level were assessed—all factors that could affect the risk of developing cancer. To assess the severity of OSA and the link to cancer development, the researchers looked at how many times the participants had partial or complete airway closure during sleep and how many times their blood oxygen levels fell during the night below 90%. The data obtained showed that the odds of cancer diagnosis were higher in women with OSA than in women without OSA and/or compared to men with OSA. Furthermore, breast cancer was most frequently encountered in women and prostate cancer in men.

Finally, a study by the University of Foggia showed that there is a relationship between the outcomes of colorectal cancer and obstructive sleep apnea [30]. The study, published in the *Journal of Oncology*, showed that the presence of OSA is an independent prognostic factor of survival. Beyond the prognostic relevance, this finding could also have therapeutic implications. A prospective cohort of 52 patients with metastatic solid tumors, 29 of whom with metastatic colorectal cancer (mCRC), was analyzed. Results showed that 34.6% of all patients and 34.5% of mCRC patients had OSA. In patients with mCRC, the presence of OSA correlated significantly with a reduced disease control rate (60% versus 94.7%).

The authors argued that three possible main mechanisms may be involved in the relationship between OSA and CRC. Hypoxia could (i) increase the expression of a transcription factor (HIF-1) involved in angiogenesis and resistance to therapy or (ii) modulate cells of the immune system, hindering anti-tumor responses and promoting some pro-tumors; or (iii) sleep disturbance could alter circadian rhythms, which are now known to be important for the expression of genes involved in cell proliferation and DNA repair.

All of these studies suggest that people who suffer from airway obstruction during sleep and whose blood oxygen saturation levels frequently drop below 90% are more likely to be diagnosed with cancer than people without OSA.

To date, no data have been published about the effect of CPAP therapy on cancer, and potentially innovative clinical trials are underway in this area. CPAP, in fact, thanks to its ability to attenuate hypoxemia and to modify the transcriptional profile of genes involved in cancer, could represent an important ally in order to improve the therapeutic response in patients with cancer and OSA; however, further studies are necessary to support this hypothesis.

## 5. Promising New Biomarkers in OSA: Micro-RNAs

The search for biomarkers that can help physicians not only diagnose OSA but also understand the pathophysiology of the disease is a research priority in the field.

In this context, microRNAs (miRNAs) have emerged as an opportunity in the era of precision medicine for the screening, diagnosis, and management of various diseases.

MiRNAs are small, non-coding RNAs (18–25 nucleotides) capable of negatively regulating gene expression at the post-transcriptional level; in fact, by binding the 3′-UTR region (UnTranslated Region) of a target mRNA, they can favor its degradation by increasing the deadenylation rate or inhibit its translation into protein [47]. In both cases, there is the silencing of the gene, the effect of which is the lack of production of the corresponding protein. These are small, antisense RNAs that bind the target messenger by complementarity of bases; in mammals, this complementarity is partial in most cases and determines a block of translation, while total complementarity occurs only rarely and induces degradation of the transcript. From a functional point of view, it has been shown that a single miRNA is able to regulate the expression of different genes and that a given gene can be regulated by different miRNAs that act synergistically with each other.

The post-transcriptional regulation of miRNA-mediated gene expression influences numerous physiological processes such as apoptosis, cell cycle, cell proliferation and differentiation, embryonic development, hematopoiesis, and adipogenesis, and it is known that alterations in the expression profile of miRNAs are closely related to onset and/or progression of diseases such as Alzheimer’s disease, fragile X syndrome, many types of cancer, and cardiovascular disease [47,48,49].

Although miRNAs are conventionally involved in the mechanisms of post-transcriptional regulation of gene expression occurring in the cytoplasmic compartment, recent studies have shown their presence also within the nucleus of human cells, where they can function as both activators and inhibitors of transcription target genes [50,51]. The miRNAs processed and matured in the cytosol, together with the associated RNAi (RNA-interference) factors, are imported into the nucleus thanks to specific proteins belonging to the karyopherin family, namely Esportin-1 (XPO1) and Importin-8 (IPO8) [52]. Once in the nucleus, miRNAs act by regulating the stability of nuclear transcripts, inducing epigenetic alterations at the level of the promoters of specific genes that determine transcriptional activation or silencing and, finally, modulating co-transcriptional alternative splicing events.

MiRNAs are involved in the pathological evolution of many diseases, as they regulate the expression of genes that can be fundamental for the physiological status of the organism. Variations in the expression levels of these small, non-coding RNAs have been observed in a large number of human diseases, including neurodegenerative diseases, liver dysfunctions, immune system disorders, cardiovascular disorders, and cancer [49,53]. Experimental evidence, in fact, shows that such pathologies can be direct consequences of alterations in cellular pathways caused by mutations in miRNA genes, of the binding sites on their targets, or in the molecular mechanisms that regulate their expression [53].

## 6. Micro-RNAs and OSA

Recent studies revealed that expression patterns of specific microRNAs are associated with OSA development and progress.

In particular, a study by Santamaria-Martos et al. showed that patients with OSA exhibit a dysregulated miRNA profile compared to non-OSA patients [54]. In this regard, it appears that intermittent hypoxia, associated with OSA, may lead to differential expression of some hypoxia-induced miRNAs. This evidence suggests the importance of miRNAs and the pathways regulated by them. An alteration in their expression could result in a deregulation of key genes and pathways that contribute to the development and progression of OSA.

To date, we know that microRNAs can be found in various biological fluids such as saliva, urine, bronchoalveolar lavage (BAL), plasma, serum, and sputum and can play a significant role as potential biomarkers especially for diagnosing disease, for monitoring the therapeutic effect of a drug, or for predicting tumor recurrence in patients treated with chemotherapy.

In particular, micro-RNAs seems to have many of the main features of a good biomarker: (i) they are stable in the circulation, (ii) they are resistant to digestion by RNase, and (iii) they are able to withstand extreme pH, high temperatures, long-term storage, and multiple freeze–defreeze cycles [55].

**Figure 2:**
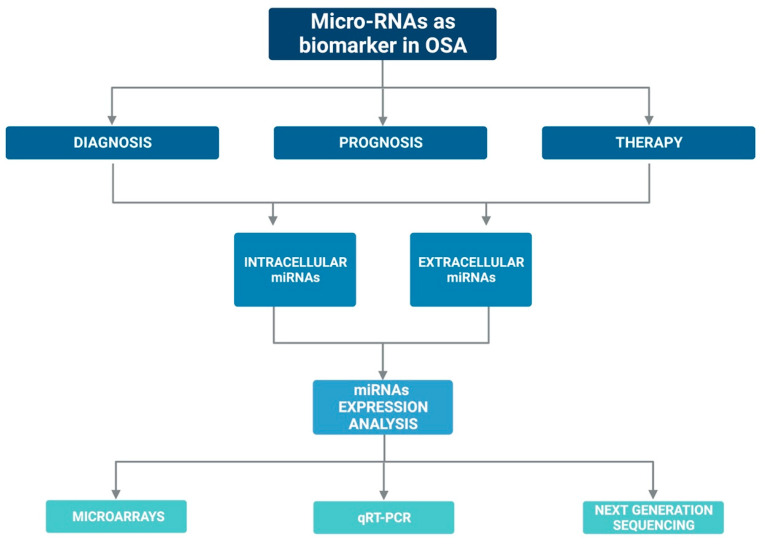
Graphic representation on the possible role of miRNAs as biomarkers in OSA.

Currently, there are several assumptions regarding their stability in the circulation: (i) miRNAs may have unique modifications, such as methylation, adenylation, and uridylation that increase their stability and thus protect them against RNases [56]; or (ii) miRNAs could be protected by encapsulation in cell-derived microvesicles [57] or by specific RNA-binding proteins [58,59]. In addition, the expression of miRNAs can be easily assessed with various laboratory methods such as quantitative q-PCR, microarray, and sequencing [60,61]. Figure 2 graphically represents the above mentioned.

Changes in miRNA expression levels are often associated with various diseases or some biological/pathological phases, such as OSA. Therefore, all these aspects make miRNAs potential diagnostic, prognostic, and therapeutic biomarkers for OSA.

Table 2 summarizes the main features and results of the microRNA and OSA studies included in this review. As part of the OSA, a study that demonstrates how the analysis of microRNAs could allow the characterization of OSA and could help provide a more accurate diagnosis of the disease was recently published by Santamaria-Martos et al. [54]. In this regard, in this study the expression profile of circulating miRNAs in patients with OSA and in non-OSA patients was examined to study the differences. The authors conducted an observational study of 230 male and female adults suspected of having OSA. All subjects underwent full PSG. The expression profile of plasma miRNAs was performed by array and validated with RT-qPCR. This analysis revealed that six miRNAs were significantly down regulated in male patients with OSA. The six miRNAs that from this first analysis were found to be differently expressed in patients with OSA were also evaluated after 6 months of treatment with CPAP. The results showed that miRNA levels were increased in patients treated for OSA compared with patients without OSA. However, only for miR-345 did they demonstrate a significant difference. This study allowed to identify a cluster of miRNAs useful for differentiating non-OSA patients and patients with OSA and to associate CPAP treatment with a change in the profile of circulating miRNAs. They demonstrated that CPAP treatment can induce changes in the miRNA profile, and therefore, microRNAs may be useful for treatment monitoring.

The same research group also published a study focusing on plasma miRNAs, in which they explored the use of a miRNA panel in patients with resistant hypertension and OSA to study their role in predicting therapeutic response to continuous positive airway pressure (CPAP) [62]. They examined a pool of miRNAs in patients with OSA and resistant hypertension, a condition often linked to the disorder, and evaluated the effect of 3 months of CPAP treatment on their blood pressure. Albeit this investigation provided evidence of miRNAs as markers in patient-guided therapy, most publications have focused on the potential role of miRNAs as diagnostic markers of disease.

One of the first studies conducted on the subject was that of Li et al. [63]. In this work, starting from serum samples and combining sequencing and a bioinformatics approach, the authors were able to identify 104 miRNAs as potential markers of pathology. To verify the results, they conducted RT-PCR analyses, which revealed that miR-107, 199-3p, and miR-485-5p were found to be downregulated, while miR-574-5p appeared upregulated in patients with OSA compared to healthy controls.

Numerous studies have been published on this topic in recent years.

Freitas et al. [64], by means of microarray analysis, examined the expression of about 2500 miRNAs in overweight adult males divided, after a PSG, into non-OSA, mild OSA, moderate OSA, and severe OSA. The results were validated by RT-PCR and showed that miR-320e and miR-1254 exhibited a gradual increase in expression in relation to the severity of OSA.

Shao H et al. studied the expression profile of a series of miRNAs in the serum of patients with obstructive sleep apnea-hypopnea syndrome, providing a theoretical basis for the search for molecular targets for the clinical diagnosis and treatment of this pathology [65].

Khalyfa A et al. conducted various studies on the use of microRNAs as potential biomarkers for patients with OSA; one of these was conducted on a population of children with OSA and obesity and led to the identification of a subset of plasma miRNAs involved in endothelial function [66,67,68].

Since, as described above, OSA is associated with multiple comorbid conditions in the scientific community, interest is growing in the exploration of biomarkers to understand the mechanisms related to the disease and improve the stratification of OSA risk.

With regard to cardiovascular complications, a recent study brought to light the role of miR-126a-3p in patients with OSA-associated hypertension, suggesting it as a new potential therapeutic target for the treatment of OSA-associated hypertension [69].

Yang X et al. likewise examined the serum miRNAs of patients with OSA, non-hypertensive and hypertensive, and in “control” subjects [70]. The miRNA profile was evaluated by microarray and validated by RT-qPCR. One group of miRNAs showed a significant difference in OSA expression compared to controls. Let-7d-5p, miR-145-5p, and miR-320b were less expressed in the non-hypertensive OSA group than in controls, while miR-26a-5p and miR-107 were less expressed in patients with hypertensive OSA than in controls. Let-7d-5p was up regulated in hypertensive patients with OSA compared to controls. These results showed how the combination of let-7d-5p and miR-145-5p could identify patients with non-hypertensive OSA, while miR-26a-5p, miR-107, and miR-126-3p could allow the identification of hypertensive OSA.

Slouka D et al., in order to improve OSA diagnostics, also conducted a study using miRNAs as potential biomarkers circulating in the blood in the diagnosis and risk assessment of cardiovascular complications [71]. Their results demonstrated that miR-499 is involved in the regulation of gene expression during hypoxia and that it could be a new diagnostic biomarker for OSA.

Turning to neurological disorders, Targa A et al. studied the profile of circulating miRNAs associated with OSA in patients with Alzheimer’s. They observed a subset of 15 miRNAs expressed differently in Alzheimer’s patients with OSA and without OSA, suggesting a plasma miRNA signature associated with the presence of OSA in Alzheimer’s patients [72].

Li et al. described the effect of atherosclerosis on the miRNA profile of patients with OSA [73]. The goal was to identify specific serum miRNAs useful as indicators of atherosclerotic disease in patients with OSA. For this purpose, they analyzed a total of 116 male and female adult patients divided according to the presence of OSA and the carotid intima-media thickness.

The miRNA profiles were first evaluated by sequencing and subsequently validated by RT-qPCR. The expression of miR-664a-3p was downregulated in OSA patients compared to controls (in all three OSA groups subdivided according to normal carotid intima-media thickness). The authors suggested the potential of miR-664-3p as a non-invasive biomarker of atherosclerosis in OSA.

Finally, Chen YC et al. investigated the anti-inflammatory role of miR-21 and miR-23 via the TLR/TNF-α signaling pathway [74]. They observed that both miRNAs were down-regulated in patients with OSA compared to patients with primary light snoring, while TNF-α gene expression was increased. These results indicate that miR-21-5p overexpression could be a future new therapy for OSA.

**Table 2:** Main studies on microRNAs in obstructive sleep apnea.

Reference	Title	Publication Year	Experimental design	Samples	Samples size	Methods	Main Findings
Sánchez-de-la-Torre M et al. [62]	Precision Medicine in Patients with Resistant Hypertension and Obstructive Sleep Apnea: Blood Pressure Response to Continuous Positive Airway Pressure Treatment.	2015	Patients with OSA and CPAP treatment	PLASMA	38	Microarray + RT-qPCR	MiR-100-5p, miR-378a-3p, and miR-486-5p predict responses to CPAP treatment in patients with OSA.
Santamaria-Martos F et al. [54]	Circulating microRNA profile as a potential biomarker for obstructive sleep apnea diagnosis.	2019	Differences between OSA and non-OSA	PLASMA	230	TLDA + RT-qPCR	Lower levels of miR-133a, miR-181a, miR-199b, miR-340, miR-345, and miR-486-3p in OSA patients compared with non-OSA.
Targa A et al. [72]	Circulating MicroRNA Profile Associated with Obstructive Sleep Apnea in Alzheimer’s Disease.	2020	Circulating microRNA profile associated with OSA in Alzheimer’s disease.	PLASMA	29	RT-qPCR	15 miRNAs are differentially expressed between OSA and non-OSA patients with AD.
Khalyfa A et al. [67]	Circulating microRNAs as Potential Biomarkers of Endothelial Dysfunction in Obese Children.	2016	Obese or non-obese children with OSA and with endothelial dysfunction or normal endothelial function.	EXOSOMES IN PLASMA	128	Microarray + RT-qPCR	MiR-125a-5p, miR-342-3p, and miR-365b-3p were identified as potential biomarkers of children with endothelial dysfunction
Khalyfa A et al. [66]	Effect on Intermittent Hypoxia on Plasma Exosomal Micro RNA Signature and Endothelial Function in Healthy Adults	2016	Human model of intermittent hypoxia.	EXOSOMES IN PLASMA	10	Microarray + RT-qPCR	Plasma exosomal micro RNAs (miRNAs) profile.
Khalyfa A et al. [68]	Circulating plasma exosomes in obstructive sleep apnoea and reverse dipping blood pressure	2020	Exosomal microRNA in untreated OSA patients with normal immersion blood pressure, reverse immersion blood pressure, and an extreme form of non-immersion.	EXOSOMES IN PLASMA	46	Microarray + RT-qPCR	Exosomes from reverse immersion blood pressure patients increased the permeability of endothelial cell tight junctions and adhesion molecule expression.
Li K et al. [63]	MicroRNA expression profiling and bioinformatics analysis of dysregulated microRNAs in obstructive sleep apnea patients.	2017	OSA patients and healthy subjects	SERUM	6	Sequencing + RT-qPCR	Different expression of miR-107, miR-199-3p, miR-485-5p, and miR-574-5 in patients with OSA and healthy controls.
Yang X et al. [70]	MiRNA expression profiles in healthy OSAHS and OSAHS with arterial hypertension: potential diagnostic and early warning markers.	2018	3 patient groups: non-OSA, non-hypertensive OSA patients, and hypertensive OSA patients.	SERUM	60	Microarray + RT-qPCR	Let-7d-5p and miR-145-5p allow the identification of non-hypertensive patients with OSA. The miR-26a-5p, miR-107, and miR-126-3p identify hypertensive patients with OSA.
Li K et al. [73]	MiR-664a-3p expression in patients with obstructive sleep apnea.	2018	Patients divided into four groups based on the presence of OSA and carotid intima-media thickness test.	SERUM	116	Sequencing + RT-qPCR	MiR-664a-3p was downregulated in patients with OSA, and non-OSA with CIMT increased compared to controls.
Shao H et al. [65]	Expression Profile Analysis and Image Observation of miRNA in Serum of Patients with Obstructive Sleep Apnea-Hypopnea Syndrome.	2021	Differential miRNAs of OSAHS-related hypertension.	SERUM	-	Bioinformatics methods	MiR-22-3p, miR-595, and miR-6856-are involved in the pathogenesis of OSAHS-related hypertension.
Freitas LS et al. [64]	Severe obstructive sleep apnea is associated with circulating microRNAs related to heart failure, myocardial ischemia, and cancer proliferation.	2020	Four groups: non-OSA, mild OSA, moderate OSA, and severe OSA	BLOOD	48	Microarray + RT-qPCR	MiR-320e and miR-1254 are associated with severe OSA.
Slouka D et al. [71]	The potential of miR-499 plasmatic level as a biomarker of obstructive sleep apnea syndrome	2021	Study of miR-1-3p, miR-133a-3p, and miR-499a-5p plasmatic levels in OSA	BLOOD	-	Reverse transcription-PCR	MiR-499 influences gene expression and could be a putative biomarker for OSA.
Chen YC et al. [74]	miR-21-5p Under-Expression in Patients with Obstructive Sleep Apnea Modulates Intermittent Hypoxia with Re-Oxygenation-Induced-Cell Apoptosis and Cytotoxicity by Targeting Pro-Inflammatory TNF-α-TLR4 Signaling.	2020	Levels of miR-21, miR-23a, and their target genes are assessed in PBMC from patients with severe OSA and 20 subjects with primary snoring (PS).	PBMC	60	RT-qPCR	Lower levels of miR-21-5p and miR-23-3p and higher levels of TNF-α both in OSA patients and in IHR-induced apoptotic monocytes.
He L et al. [69]	miR-126a-3p targets HIF-1α and alleviates obstructive sleep apnea syndrome with hypertension.	2020	Role of miR-126a-3p in OSA-hypertension.	Sprague–Dawley rats and rat aortic smooth muscle cells (A7r5)	24 rats	RT-qPCR	MiR-126a-3p is a novel potential therapeutic target for the treatment of OSA-hypertension.

## 7. Strengths and Limitations

All of the above studies show microRNAs as small but very important molecules discovered only a few years ago that play a key role in regulating the expression of various genes simultaneously in response to different biological stimuli. MicroRNAs are implicated in many physiological processes such as cell growth or differentiation as well as several pathological processes including cancer [75]. Their flexibility gives microRNAs great potential as therapeutic molecules, and numerous clinical trials are testing their use in diseases such as sleep-disordered breathing and cancer.

In almost all the mentioned studies, a variation of the expression of specific miRNAs, both free and packaged inside extracellular vesicles, is shown in OSA patients [54,62,63,64,65,66,67,68,69,70,71,72,73,74].

Almost all of them used real-time PCR as the analysis technique in association with more advanced techniques such as microarray or sequencing. Many studies showed a difference in miRNA expression before and after CPAP treatment; others, instead, showed a different miRNA expression in association with some comorbidities. However, in all studies, miRNAs acted as excellent biomarkers, easily measurable with standard procedures and associated with the disease and with different expression following treatment. In addition, various biological samples were used, such as blood, serum, plasma, or PBMC, for example. This to point out that microRNAs can be found in almost all biological fluids, a feature that makes them excellent non-invasive biomarkers for the diagnosis, prognosis, and treatment of various diseases.

In summary, all the studies listed demonstrate how microRNAs may have enormous potential to function as clinical diagnostic biomarkers for many diseases. They play a fundamental role in the pathogenesis of many diseases including OSA. However, more in-depth research and confirmatory experiments are needed to establish the role of these miRNAs as biomarkers.

## 8. Conclusions

Based on what has been revealed about OSA, it is now clear that the diagnosis, management, and therapy must be personalized, evaluating the various options available depending on the patient’s “phenotype”. In this regard, new stratification, therapy, and treatment response strategies need to be explored.

In order to develop a personalized diagnostic-therapeutic approach, there is therefore an urgent need for a “phenotyping” of the patient with OSA, which must take into account the following:

(i) The extreme variability of the clinical presentation of the patient with obstructive sleep apnea and must reflect the anthropometric characteristics, the associated comorbidities, and the environmental factors involved, such as cigarette smoking and sedentary habits;
(ii) New biomarkers useful for disease stratification and treatment response.

This review therefore aims to focus on the possibility of using, in association with the apnea-hypopnea index detected by a polysomnographic study, new markers such as micro-RNAs for the definition of disease severity and as indicators of response to therapy.

In fact, microRNAs are important regulators of gene expression, and numerous studies have shown their involvement in the development and progression of many diseases including cancer.

It is known that many miRNAs are directly involved in the development of OSA and that, in general, miRNAs are involved in the formation of tumors.

Inasmuch as the cellular response to hypoxia involves the activation of various transcriptional regulators involved in inflammation, tumor invasion, angiogenesis, cell cycle block, and apoptosis, a better understanding of all these closely related mechanisms and the identification of miRNAs sensitive to hypoxia could prove fruitful in the search for new therapeutic targets and in the search for new and more effective anti-tumor therapies.

Notwithstanding, to date, there are insufficient data to elect these small, non-coding RNA molecules as diagnostic biomarkers in diseases. Numerous studies are still needed in order to develop miRNA-based therapies that can be useful in clinical practice.

Therefore, this review provides an overview of the functional relevance of miRNAs in OSA and suggests them as potential biomarkers and as a future direction in therapy.

## Figures and Tables

**Table 1:** Main symptoms and signs of OSA [11].

SYMPTOMS	
*TYPES*	- Snoring - Feeling of suffocation during sleep - Respiratory pause or reported apnea - Not-restful sleep - Daytime drowsiness - Dry mouth upon waking
*FREQUENT*	- Difficulty concentrating - Recent memory drop - Morning headache - Restlessness during sleep - Involuntary movements - Need for nocturnal urination - Reduction of daytime performance - Night sweats
*LESS COMMON*	- Gastroesophageal reflux Laryngospasm Reduced libido Nocturnal enuresis
**SIGNS**	
*BODY MASS INDEX—BMI*	>29
*NECK CIRCOMFERENCE*	>43 cm (men) >41 cm (women)
*CRANIAL-FACIAL DYSMORPHISMS*	Cause a reduction in the caliber of the upper airways
*PHARYNGEAL ANOMALIES*	Cause a reduction in the caliber of the upper airways

## Data Availability

The data presented in this study are available on request from the corresponding author.
